# Mixing thermodynamics and electronic structure of the Pt_1−*x*_Ni_*x*_ (0 ≤ *x* ≤ 1) bimetallic alloy

**DOI:** 10.1039/c9ra02320h

**Published:** 2019-05-30

**Authors:** Louise M. Botha, David Santos-Carballal, Umberto Terranova, Matthew G. Quesne, Marietjie J. Ungerer, Cornelia G. C. E. van Sittert, Nora H. de Leeuw

**Affiliations:** Laboratory for Applied Molecular Modelling, Research Focus Area: Chemical Resource Beneficiation, North-West University 11 Hoffman Street Potchefstroom 2520 South Africa; School of Chemistry, Cardiff University Main Building, Park Place Cardiff CF10 3AT UK SantosCarballalD@cardiff.ac.uk DeLeeuwN@cardiff.ac.uk +44 (0)29 2087 4715 +44 (0)29 2087 0658; Materials Modelling Centre, School of Physical and Mineral Sciences, University of Limpopo Private Bag x 1106 Sovenga 0727 South Africa; Department of Earth Sciences, Utrecht University Princetonplein 8A 3584 CD Utrecht The Netherlands

## Abstract

The development of affordable bifunctional platinum alloys as electrode materials for the oxygen reduction reaction (ORR) and oxygen evolution reaction (OER) remains one of the biggest challenges for the transition towards renewable energy sources. Yet, there is very little information on the optimal ratio between platinum and the transition metal used in the alloy and its impact on the electronic properties. Here, we have employed spin-polarised density functional simulations with long-range dispersion corrections [DFT–D3–(BJ)], to investigate the thermodynamics of mixing, as well as the electronic and magnetic properties of the Pt_1−*x*_Ni_*x*_ solid solution. The Ni incorporation is an exothermic process and the alloy composition Pt_0.5_Ni_0.5_ is the most thermodynamically stable. The Pt_0.5_Ni_0.5_ solid solution is highly ordered as it is composed mainly of two symmetrically inequivalent configurations of homogeneously distributed atoms. We have obtained the atomic projections of the electronic density of states and band structure, showing that the Pt_0.5_Ni_0.5_ alloy has metallic character. The suitable electronic properties of the thermodynamically stable Pt_0.5_Ni_0.5_ solid solution shows promise as a sustainable catalyst for future regenerative fuel cells.

## Abstract 2

Density functional theory simulations complemented by force-field based calculations show that the bimetallic Pt_0.5_Ni_0.5_ equilibrium composition is highly ordered at room conditions.

## Graphical abstract

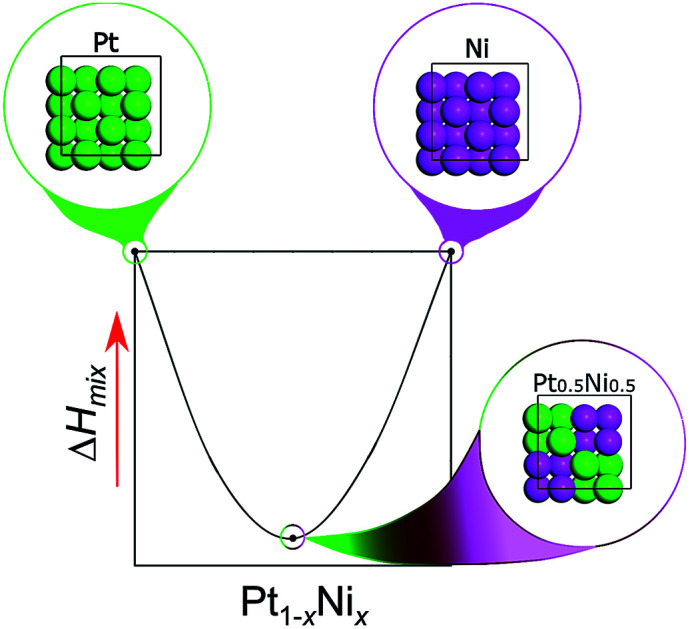

## 1 Introduction

Clean and renewable energy resources are needed to decrease pollution and climate change, whilst also keeping up with increasing energy demand. To this end, electrocatalysts are crucial components of regenerative fuel cells.^1–3^ In these devices, both the forward oxygen evolution reaction (OER), producing the clean hydrogen energy vector through water electrolysis, and the reverse oxygen reduction reaction (ORR), generating energy *via* the recombination of oxygen and hydrogen into water, take place following different mechanisms. However, the future development of regenerative fuel cells is hindered by the large over-potential exceeding the 1.23 eV required by the oxygen producing anode and slow reaction kinetics, which reduces the efficiency of the process.^4–6^ Pt-based electrocatalysts exhibit excellent activity for the ORR reaction,^7–14^ but poor performance for the OER reaction, while state of the art IrO_2_ and RuO_2_ OER electrocatalysts are only moderately active towards the ORR reaction.^2^ Electrocatalysts which combine two of these precious metals, Pt, Ir and Ru, show reasonable activity towards both the ORR and the OER reactions, but their use is limited due to low abundance and high cost.^15,16^ Therefore, the development of efficient bifunctional electrocatalysts which can be used for both the ORR and the OER reactions remains a challenge.

Previous work has reported the enhanced bifunctional electrocatalytic activity of a Pt/Ni alloy.^3^ However, the relationship between the equilibrium composition of the mixture, the distribution of the metal atoms and the electronic structure of the alloy are not yet fully understood. Insights into all these properties are, however, essential if we are to utilise these types of bimetallic materials as fuel cell catalysts for the ORR and OER reactions. In this paper, we have used density functional theory (DFT) calculations to study the mixing thermodynamics of the solid solution with Pt and Ni as end members, as well as the electronic and thermodynamic properties for the equilibrium composition.

## 2 Computational details

We have used DFT calculations as implemented in the Vienna ab initio simulation program (VASP)^17–20^ to calculate the structures and energies of the Pt–Ni solid solution. The generalised-gradient approximation (GGA) functional developed by Perdew, Burke, and Ernzerhof (PBE),^21,22^ corrected with the semiempirical D3 approach of Grimme with the Becke–Johnson (BJ) damping,^23–25^ was used for the simulations. The interaction between the core and valence electrons, defined as those in the 3d4s levels for Ni and 5d6s for Pt, was described with the projected augmented-wave (PAW)^26^ method in the implementation of Kresse and Joubert.^27^ A *Γ*-centred Monkhorst–Pack grid^28^ of 11 × 11 × 11 *k*-points was used to carry out the integrations in the reciprocal space of the primitive unit cells. The *k*-point grids for larger supercells were chosen in such a way that a similar spacing of points in the reciprocal space was maintained. To improve the convergence of the Brillouin-zone integrations, the electronic partial occupancies were determined using the Methfessel–Paxton order 1 smearing, with a width for all calculations set at 0.2 eV. However, the tetrahedron method with Blöchl corrections was used in static simulations to obtain very accurate total energies as well as all the electronic and magnetic properties. The kinetic energy cut-off was fixed at 400 eV for the plane-wave basis set expansion of the Kohn–Sham (KS) valence states, which was large enough to avoid Pulay stress. The atomic charges and magnetic moments were analysed using the Bader partitioning.^29–34^

The crystal structure of both Pt and Ni metals is face-centred cubic (*fcc*), which belongs to the *Fm*3̄*m* space group (No. 225).^35,36^ The primitive cubic unit cell of the pure metals contains 4 atoms distributed in the Wyckoff 4a positions. Pt is a closed shell paramagnetic metal, while Ni is a ferromagnetic material with a Curie temperature of *T*_C_ = 624 K.^37^ For the pure metals and solid solutions, all the calculations were spin-polarised and the initial spin moments were set parallel for the Ni atoms.

The internal coordinates, lattice parameters, atomic spin moments and cell shape were all allowed to change during geometry optimisations. Using this methodology, we have calculated a lattice parameter *a* for the bulk unit cell of the pure Pt and Ni end member phases of 3.924 and 3.473 Å, in excellent agreement with the experimental values of 3.923^35^ and 3.528 Å,^36^ respectively. Taking into account the performance of our DFT setup for predicting the lattice parameters of the pure metal phases, we are confident of our predictions for the solid solution containing various Pt/Ni compositions.

In addition to the DFT calculations, atomistic simulations based on interatomic potentials (IP) were also carried out using the General Utility Lattice Program (GULP).^38,39^ The Sutton and Chen interatomic potentials were used to calculate the energies and structures of the pure Pt and Ni metals.^40^ The interatomic potentials for the alloy were obtained using the standard geometric and arithmetic average rules.^41^ The complementary use of a less compute-intensive method allowed us to sample larger simulation cells in our mixing studies.

We have used the site occupancy disorder (SOD) code^42^ to study a series of substitutions of Pt by Ni in the 1 × 1 × 1 and 2 × 2 × 1 supercells of the *fcc* unit cell, containing 4 and 16 sites, respectively. This program constructs the full configurational spectrum for each composition of the supercell and then uses the symmetry operators of the parent structure to separate the symmetrically inequivalent configurations. Note that the 2 × 2 × 1 supercell breaks the cubic symmetry of the unit cell, which affects the atomic interactions with the neighbouring cells in the *c* direction with respect to the *a* and *b* directions. However, the fully symmetric 2 × 2 × 2 supercell, has a prohibitively large computational cost when considering all inequivalent substitutional configurations, even when employing atomistic simulations.

## 3 Results and discussion

### 3.1 Configurational entropy of mixing

Firstly, we discuss the full-disorder or maximum configurational entropy (*S*_max_), which we have defined as the number of ways the Pt and Ni atoms pack together in the solid solution for a given composition in the high temperature limit. The maximum configurational entropy for a finite simulation cell is obtained from^43^1
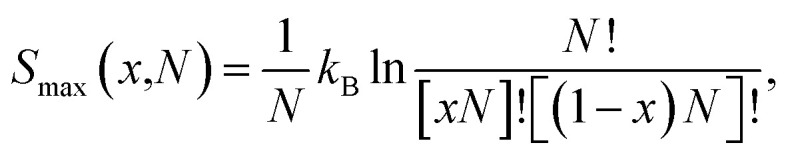
where *N* is the number of formula units in the supercell, *k*_B_ is the Boltzmann constant and *x* is the Ni mole fraction in the alloy. The ideal full-disorder entropy (*S*_ideal_)^43^ as the cell size approaches infinity is calculated as2
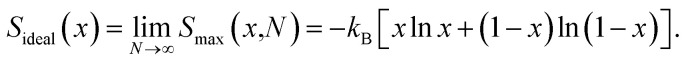


**Fig. 1:**
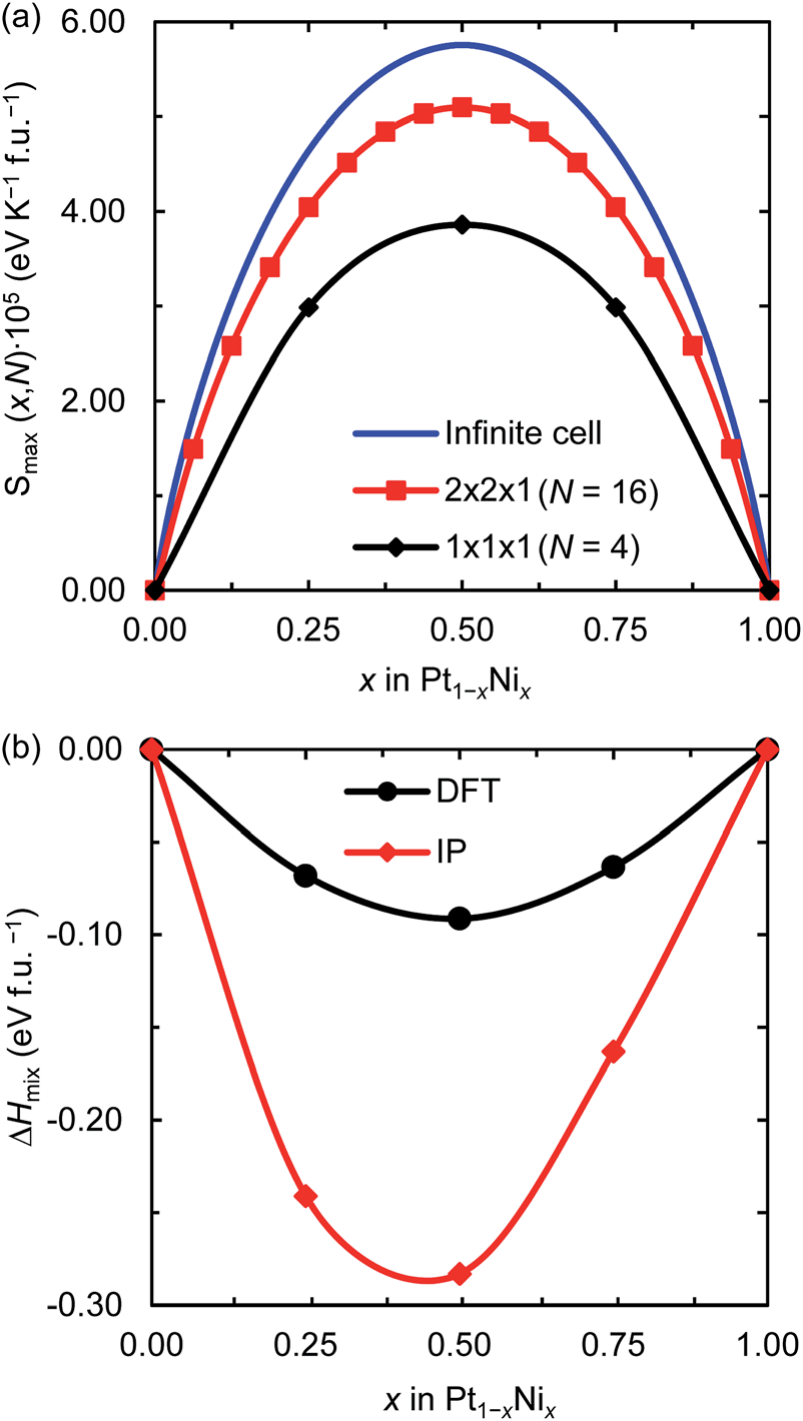
(a) Maximum configurational entropy (*S*_max_) for different number of f.u. (*N*) and (b) mixing enthalpies (Δ*H*_mix_) for the 1 × 1 × 1 supercell both per formula unit of Pt_1−*x*_Ni_*x*_ and as a function of the Ni mole fraction (*x*). Δ*H*_mix_ was calculated using DFT and IP-based simulations.

Fig. 1(a) displays the maximum entropy values for different simulation cell sizes and Ni contents. As expected from eqn (2), the largest full-disorder entropies are reached when both Pt and Ni atoms have equal concentrations, starting at *S*_max_ = 0 for the pure metal phases. For the smallest 1 × 1 × 1 supercell, used for our DFT calculations, the maximum entropy varies significantly from the ideal value for any Ni mole fraction. For example, the calculated high temperature entropies for the simulation cell containing 4 f.u. are below the full-disorder values by ∼36 and ∼33% at *x* = 0.25 and 0.50, respectively. Given the cubic symmetry of the primitive unit cell, we chose to double any two axes to form the 2 × 2 × 1 supercell used in the atomistic simulations. The resulting full-disorder configurational entropies improve significantly with respect to the cell containing 4 f.u. Configurational entropies have a slow rate of convergence with respect to the size of the simulation cell, in agreement with previous reports.^44^ Although not fully converged, the maximum entropies are still useful to evaluate the level of disorder in solid solutions at equilibrium conditions.

### 3.2 Enthalpy of mixing

To gain insight into the thermochemistry of Ni incorporation in Pt, we have calculated the enthalpy of mixing (Δ*H*_mix_) as^43^

3 Δ*H*_mix_ = *E*(Pt_1−*x*_Ni_*x*_) − (1 − *x*)*E*(Pt) − *xE*(Ni),

where *E*(Pt_1−*x*_Ni_*x*_), *E*(Pt) and *E*(Ni) are the total energies of the Pt_1−*x*_Ni_*x*_ mixed system and the pure Pt and Ni metals, respectively. In particular, *E*(Pt_1−*x*_Ni_*x*_) corresponds to the average^43,45,46^4
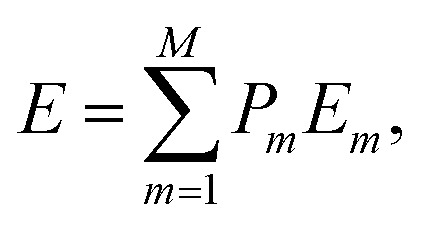
where *P*_*m*_ and *E*_*m*_ are the occurrence probability and the energy of configuration *m*, respectively, in the full spectrum containing *M* inequivalent configurations. We recall that the configurational probabilities follow the Boltzmann distribution5
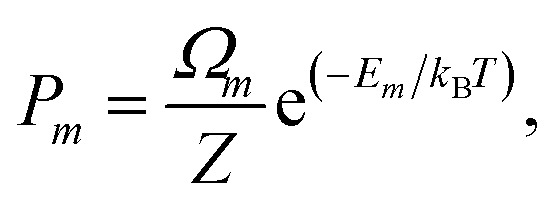
where *Ω*_*m*_ is the degeneracy of the inequivalent configuration *m* and *Z* is the configurational partition function6
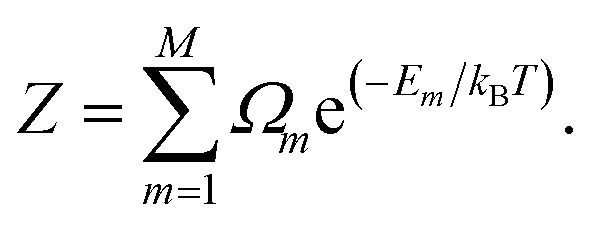


At this point it is worth mentioning that the configurational space of the 1 × 1 × 1 supercell contains only one symmetrically inequivalent configuration for each of the 5 possible Ni mole fractions. Thus, the corresponding energetics of the mixing does not depend on temperature, as described in eqn (4).

Fig. 1(b) shows the DFT and IP enthalpy of mixing as a function of the Ni mole fraction *x* for the 1 × 1 × 1 supercell. The calculated Δ*H*_mix_ values for the partially disordered alloy are negative for any Ni concentration, indicating that the formation of the Pt_1−*x*_Ni_*x*_ solid solution is an exothermic process. Our DFT (IP) simulations suggest that there is a small asymmetry in the enthalpy of mixing of the disordered system as Ni incorporation in Pt is 0.005 (0.078) eV per formula unit (f.u.) more exothermic than Pt incorporation in Ni. Although the IP method predicts larger enthalpies of mixing than the DFT approach, especially for *x* = 0.5, they describe the same behaviour for the thermochemistry of the Pt–Ni solid solution. In particular, they both predict Pt_0.5_Ni_0.5_ to be the equilibrium composition, which has also been observed in a computational study into the ordered and disordered special quasi-random structured Pt_1−*x*_Ni_*x*_ solid solution.^47^

### 3.3 Probability distribution

**Fig. 2:**
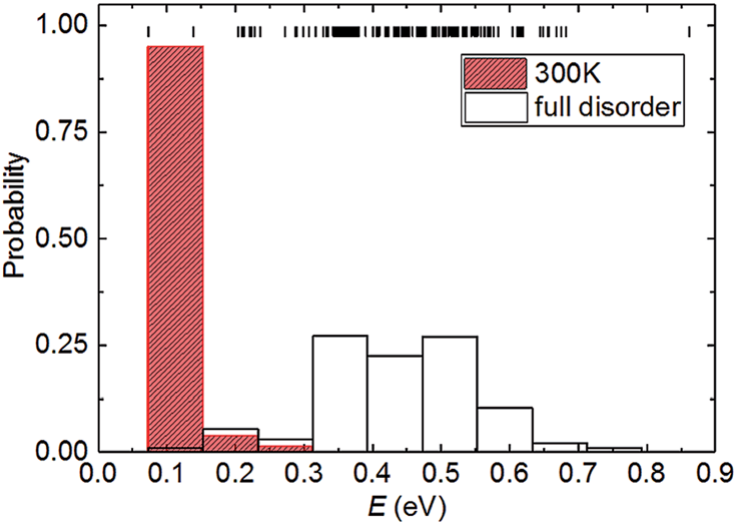
Partially and fully disordered probability distribution of the energies (*E*) calculated for the configurations with Ni composition *x* = 0.50 in the 2 × 2 × 1 supercell. The short vertical lines represent the energy values in the configurational spectrum. The partially disordered probability distribution was calculated using a Boltzmann-modulation at 300 K.

We now consider the relationship between the configurational energy and the probability of the partially and fully disordered system with Pt_0.5_Ni_0.5_ composition. In Fig. 2, we have plotted the probability distributions of the 153 inequivalent configurations for the 2 × 2 × 1 supercell at Ni mole fraction *x* = 0.50. Our atomistic simulations clearly indicate that there is a 0.7 eV f.u.^−1^ difference between the low- and high-end energy configurations for the Pt_0.5_Ni_0.5_ composition. At high temperatures, where the solid solution is fully disordered, we have found that the degeneracy weighting leads to a normal probability distribution where those configurations with intermediate energies of ∼0.45 eV f.u.^−1^ are favoured. However, upon equilibration at lower temperatures the lowest-energy configurations have a high weighting while the probabilities decrease with the energy of the configurations. For example, we have predicted the probability distribution for the partially disordered solid solution with Pt_0.5_Ni_0.5_ composition at 300 K to represent the ambient conditions. Fig. 2 shows that when considering temperature effects, the probability of occurrence vanishes for 150 configurations. The remaining three configurations, to which we will refer as “*A*”, “*B*” and “*C*” account for 3.7, 47.6 and 47.3%, respectively, of the total probability at 300 K. The ground state configurations *B* and *C* strongly suggest that Pt_1−*x*_Ni_*x*_ bimetallic alloys prefer to form an ordered rather than a random distribution of atoms at 300 K, a preference that is reduced at higher temperatures. This effect is not surprising, as previous simulations of the Ca_1−*x*_Mn_*x*_CO_3_ system have also shown that full disorder is unlikely at equilibrium conditions, suggesting a significant deviation from a random distribution of cations.^43^ We do not consider configuration *A* for further analysis because of its low probability, which makes it very unlikely to occur in real samples.

### 3.4 Ground state configurations

**Fig. 3:**
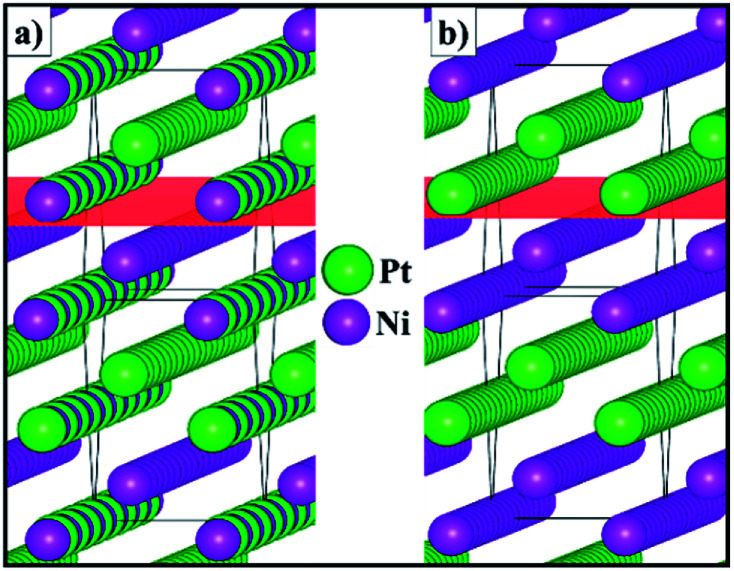
Schematic representation of configurations (a) *B* and (b) *C* for the 2 × 2 × 1 supercell of the solid solution with the Pt_0.5_Ni_0.5_ composition. The (110) plane is represented in red.

Fig. 3 shows the schematic representation of the lowest energy configurations *B* and *C* identified for the 2 × 2 × 1 supercell. Although the Pt and Ni atoms do not segregate in two separate phases, our atomistic simulations provide evidence of a preferential ordering pattern. For configuration *B*, we have found three different alternating atomic planes perpendicular to the [110] direction which are represented as a layer of Pt, followed by a mixed layer of Pt/Ni and terminated by a single layer of Ni. Configuration *C* also exhibits the ordering pattern in the [110] direction, where every other mixed layer is replaced by a plane containing only one type of atom, leading to alternating double layers of Pt and Ni metals. The atomic orderings that we have found for configurations *B* and *C* along the [110] direction are equivalent to the surface segregation patterns predicted computationally^48^ and later confirmed experimentally,^49–53^ which were shown to have a strong effect on the catalytic properties of the alloy.

### 3.5 Density of states

**Fig. 4:**
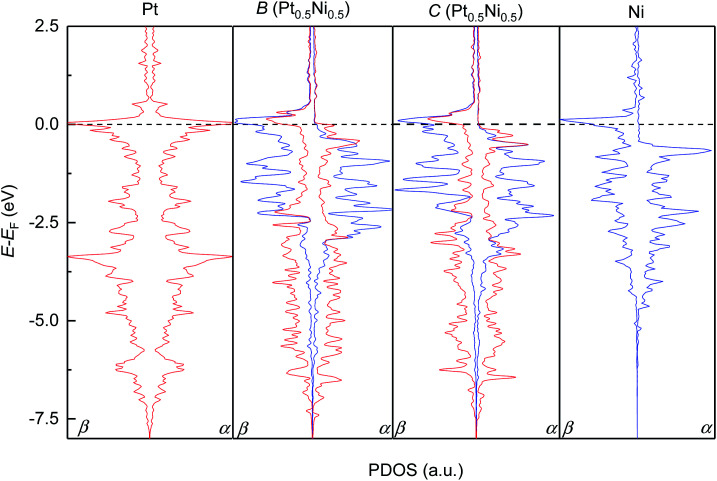
Atomic projections of the spin-decomposed density of states (PDOS) for the d-electrons of the pure Pt and Ni metals (left and right panels) and configurations *B* and *C* of the solid solution with the Pt_0.5_Ni_0.5_ composition (middle panels). α and β stand for the majority and minority channel of the spins, respectively.

We subsequently modelled the electronic structures and magnetic properties of the highest probability configurations *B* and *C* of the Pt_0.5_Ni_0.5_ solid solution and compared them to those of the parent metals. Fig. 4 depicts the projected density of states (PDOS) for the d-electrons of the pure metals Pt and Ni and configurations *B* and *C*. As expected, we have found that Pt metal is non-magnetic and has a very symmetric PDOS in both channels of the spins due to the equal population of electrons with α- and β-spins within the full 5d^10^ level. The occupied d levels of Pt appear between −7.5 eV and the Fermi level (set to 0.0 eV), with prominent bands at −3.0 and 0.0 eV. In contrast, Ni has an asymmetric PDOS, especially around the Fermi level, due to its incomplete 3d^9^ level. The valence band in the majority channel of the spins in Ni spans from −5.0 to −0.5 eV, while states in the β channel are shifted approximately 1 eV towards the Fermi level. The Ni levels with the largest PDOS are located at the valence band maximum for the α channel and at the conduction band maximum for the β channel. Despite the slightly different Pt and Ni metal arrangement, our DFT simulations suggest that the electronic structures of configurations *B* and *C* are very similar. The itinerant electron magnetism between the Pt and Ni atoms induces an asymmetry in the PDOS, which can be understood as an electronic delocalisation due to the strong interaction between the d electrons of the two metals. Furthermore, the Pt and Ni bands are strongly hybridised, particularly around the Fermi level and at −2.0 eV for the two channels of the spins of configurations *B* and *C*. The top and bottom edges of the d valence bands for the Pt and Ni atoms run within similar energy values in the solid solution and in the pure phases. We have found that the PDOS of the conduction bands are negligible for the pure metals Pt and Ni as well as configurations *B* and *C* of Pt_0.5_Ni_0.5_, apart from the bands crossing the Fermi level.

### 3.6 Electronic band structure

**Fig. 5:**
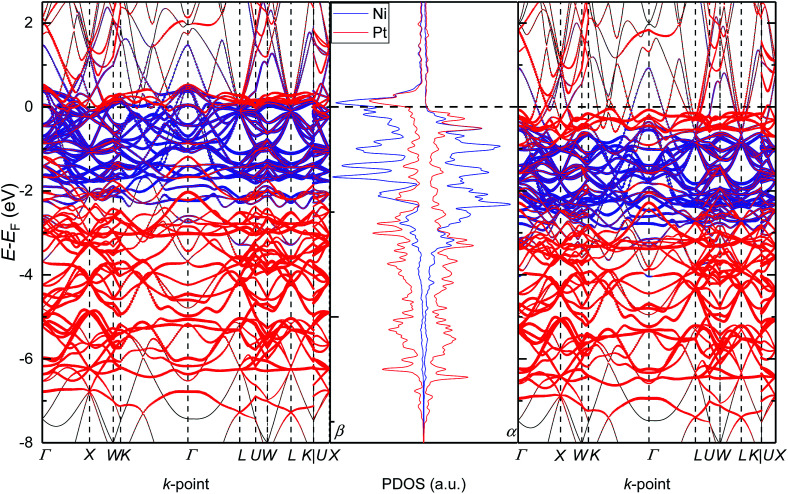
Electronic density of states and band structure for configuration *B* of the solid solution with the Pt_0.5_Ni_0.5_ composition. α and β stand for the majority and minority channel of the spins, respectively.

To further analyse the electronic properties of the solid solution with the Pt_0.5_Ni_0.5_ composition, we have also plotted in Fig. 5 the band structure of configuration *B* alongside its PDOS. We have chosen the path suggested by Setyawan *et al.*^54^ to connect the high symmetry points of the first Brillouin zone. We have confirmed the lack of band gap for any of the spin channels and their asymmetry, in agreement with the PDOS. The number of bands crossing the Fermi level (*E*_F_) in the minority spin channel is larger than in the majority spin channel, suggesting a larger contribution of the β electrons to the metallic properties of the alloy. The α spin contribution to the band structure is spread between −0.5 and −2.9 eV for Ni, while the Pt states are mostly below and above the Ni bands down to −7.40 eV and up to the Fermi level, respectively. The β spin channel shows similar features to the opposite channel but are all displaced towards the Fermi level by approximately 1.0 eV. Interestingly, the band structure of configuration *B* clearly indicates the origin of the hybridisation between the d states of both atoms as the Pt d levels have a larger contribution than the Ni ones at the lines connecting the high-symmetry points *W*–*K*, *U*–*W* and *U*–*X* of the first Brillouin zone in both spin channels.

### 3.7 Atomic charges and spin moments

Table 1 summarises the atomic charges (*q*) and atomic spin moments (*m*_s_) for the pure metallic phases as well as the two major configurations of the solid solution with the equilibrium composition. For the two metals, we have confirmed that the atoms are charge-neutral. The Pt atom is not magnetic and the Ni has 0.630 *μ*_B_ per atom, comparing well with the experimental value.^55^ We have found that Pt becomes magnetic upon insertion of the Ni atoms in the solid solution as it gains electrons in its 6s level.^56^ The atomic quantities are very similar for Pt_0.5_Ni_0.5_, albeit that the Pt magnetic moment is marginally lower in configuration *B* than in *C*. The total spin magnetisation of saturation (*M*_S_) is 1.07 and 1.11 *μ*_B_ f.u.^−1^ for configurations *B* and *C*, while the Ni atoms lose ∼30% of their atomic spin moments to Pt.

**Table 1:** Atomic charges (*q*) and atomic spin moments (*m*_s_) for the pure Pt and Ni metals as well as for configurations *B* and *C* of the solid solution with the Pt_0.5_Ni_0.5_ composition. Atomic properties were calculated by means of a Bader analysis

Phase	Configuration	Atom	*q* (e per atom)	*m*_s_ (*μ*_B_ per atom)
Pt	—	—	0.00	0.00
Ni	—	—	0.00	0.63
Pt_0.5_Ni_0.5_	*B*	Pt	−0.23	0.33
		Ni	0.23	0.74
	*C*	Pt	−0.23	0.35
		Ni	0.23	0.76

## 4 Conclusions

In this study, we have reported the mixing thermodynamics along with the geometrical, electronic and magnetic structures for the Pt_1−*x*_Ni_*x*_ solid solution. We have modelled all symmetrically inequivalent configurations for five Ni concentrations by using the 1 × 1 × 1 supercell, and for the equilibrium composition in the 2 × 2 × 1 supercell. The equilibrium Ni mole fraction *x* determined for the Pt_1−*x*_Ni_*x*_ system shows that Pt and Ni have a 1 : 1 ratio in the solid solution, in agreement with the available experimental data. The solid solution with the Pt_0.5_Ni_0.5_ composition is highly ordered and up to at least 300 K does not experience segregation of the atomic species, as the only two major symmetrically inequivalent configurations each have approximately a 50% probability of occurring. The Pt and Ni atoms in the two major configurations *B* and *C* tend to form similar ordered patterns in the [110] direction. The projected density of states for the Ni metal is asymmetric due to its magnetic nature, while the non-magnetic Pt is symmetric. We have found that both configurations have metallic properties. The d levels of the two atoms cross the Fermi level, but the Pt contribution is only larger in those lines connecting the high-symmetry points *W*–*K*, *U*–*W* and *U*–*X* of the first Brillouin zone. The Pt atoms gain negative charge following the formation of the solid solution, thereby allowing them to become magnetic. The calculated lattice parameters, atomic charges and spin moments are in excellent agreement with experiments for the pure phases. Future work will involve the simulation of the electrocatalytic activity on the major Pt_0.5_Ni_0.5_ surfaces for the two most stable configurations.

## Conflicts of interest

There are no conflicts to declare.

## Supplementary Material
